# Intergenerational plasticity aligns with temperature-dependent selection on offspring metabolic rates

**DOI:** 10.1098/rstb.2022.0496

**Published:** 2024-02-26

**Authors:** Amanda K. Pettersen, Neil B. Metcalfe, Frank Seebacher

**Affiliations:** ^1^ School of Life and Environmental Sciences, The University of Sydney, Sydney, New South Wales 2006, Australia; ^2^ School of Biodiversity, One Health & Veterinary Medicine,, University of Glasgow, Glasgow, G12 8QQ, UK

**Keywords:** development, energy, maternal effects, metabolism, parental investment, reproductive investment

## Abstract

Metabolic rates are linked to key life-history traits that are thought to set the pace of life and affect fitness, yet the role that parents may have in shaping the metabolism of their offspring to enhance survival remains unclear. Here, we investigated the effect of temperature (24°C or 30°C) and feeding frequency experienced by parent zebrafish (*Danio rerio*) on offspring phenotypes and early survival at different developmental temperatures (24°C or 30°C). We found that embryo size was larger, but survival lower, in offspring from the parental low food treatment. Parents exposed to the warmer temperature and lower food treatment also produced offspring with lower standard metabolic rates—aligning with selection on embryo metabolic rates. Lower metabolic rates were correlated with reduced developmental and growth rates, suggesting selection for a slow pace of life. Our results show that intergenerational phenotypic plasticity on offspring size and metabolic rate can be adaptive when parent and offspring temperatures are matched: the direction of selection on embryo size and metabolism aligned with intergenerational plasticity towards lower metabolism at higher temperatures, particularly in offspring from low-condition parents. These findings provide evidence for adaptive parental effects, but only when parental and offspring environments match.

This article is part of the theme issue ‘The evolutionary significance of variation in metabolic rates’.

## 1. Introduction

Selection on life-history strategies can drive the evolution of metabolic rate, which represents the energetic cost of living [1,2]. Metabolic rates expressed during early life are associated with key life-history traits: individuals with faster minimal metabolic rates have faster developmental and growth rates, earlier onset of reproduction and shorter lifespan than slow metabolic phenotypes [3,4]. The majority of ectotherms undergo embryonic development in eggs, with a finite amount of energy reserves available to sustain cell division, differentiation and maintenance costs until post-hatching feeding [5]. Hence, variation in metabolic rates will also determine how quickly energy reserves are depleted for these species, with important consequences for survival [6]. It might be expected, therefore, that selection should act to suppress minimal rates of metabolism to conserve energy, yet variation in metabolism is ubiquitous—varying by up to threefold, even after accounting for embryo size and developmental temperature [7]. Furthermore, selection for a fast pace of life may mediate the expression of higher metabolic rates [8] that can be beneficial in high competition environments [9]. Investigating the interplay between metabolic rates and survival—and the environmental dependence of this relationship—is crucial for understanding the potential adaptive capacity of variation in metabolic rates [10].

Metabolic rates have been studied for over a century [11], yet the adaptive potential of this variation in metabolism remains unclear [12]. Mixed evidence shows that metabolism is sometimes under selection (e.g. [13–15]) and is somewhat heritable [16–18] and repeatable [19,20], suggesting that the fitness consequences of slow and fast metabolic rates are context-dependent [21,22]. It is unresolved whether metabolism has evolved as a driver or simply a by-product of the pace of life. However, metabolic rates (often measured as oxygen consumption or carbon dioxide production) reflect the energy use of an organism, so that measures of metabolic rate are meaningful in linking the physiology of an individual with its life history. Metabolic rates are not fixed across ontogeny however, and within-generation acclimation can act to downregulate metabolism under low food availability [23]. While this metabolic suppression may slow the pace of life, it can also facilitate survival under stressful conditions [24]. If there is a causal relationship between metabolism and the pace of life, then context-dependent selection may drive a correlated suite of responses [8]. Elucidating the links between metabolic rate, the pace-of-life and its fitness consequences is critical for understanding the capacity for organisms to respond to changing environments [25].

The environment that a parent experiences can shape the phenotype of their offspring, known generally as parental effects [26]. Epigenetic inheritance across two or more generations (termed inter- and trans-generational plasticity, respectively) [27] can be adaptive or maladaptive—acting as either a buffer or conduit to the effects of environmental stress [28]. Adaptive parental effects arise when parents anticipate and respond to environmental cues, to produce shifts in their offspring's phenotype that maximize their fitness in the offspring environment [26]. For example, when exposed to cool temperatures, mothers tend to produce larger offspring [29], leading to enhanced offspring survival in that same environment [30,31]. Alternatively, under a bet-hedging strategy, parents in stressful or unpredictable environments increase variance in their offspring phenotypes, with variable consequences for offspring fitness, but overall enhancing parental fitness [32]. If intergenerational plasticity is adaptive such that it confers fitness benefits for offspring, then shifts in parental provisioning should be in line with selection on offspring traits. Conversely, increased variance in parental investment that does not enhance offspring fitness consistently may be indicative of a bet-hedging strategy to maximize parental fitness. Overall trends across studies show that intergenerational plasticity on offspring phenotype is generally weak compared with the direct effects of the offspring environment [33,34], and caution needs to be exercised when inferring the adaptive value of trait plasticity [35,36]. Nonetheless, epigenetic inheritance is an important source of phenotypic variation. In particular, when environmental conditions are correlated between generations, maternal effects can account for up to half of the phenotypic variation within populations as additive genetic effects [37,38].

Adaptive parental effects are thought to evolve in changing but predictable environments to enhance offspring fitness [39], however formal selection analyses are lacking. Selection is the phenotypic covariance between fitness and a trait [40], yet most transgenerational studies have reported the effect of parental environment on an aspect of offspring performance that may trade off with actual fitness [41]. Selection analysis uses multiple regression of individual relative fitness on traits of interest to estimate standardized linear and nonlinear selection coefficients [42]. Used in combination with experimental manipulation of environmental predictability across generations, selection analysis can reveal the relative scope for evolutionary change on an offspring trait. If parents can anticipate the environment their offspring will experience, and provision accordingly, then selection on offspring metabolic rates should align with shifts in offspring investment. However, in cases where the offspring environment differs unpredictably from the parental environment, the direction, form and strength of selection may not align with the mean and variance of offspring phenotypes that parents produce. Selection analysis cannot clarify whether shifts in offspring phenotype in response to parental environment have evolved in response to selection (i.e. whether they are due to genetic or epigenetic causes), however it does provide a meaningful first step to understanding whether intergenerational plasticity is likely to be adaptive in a given environment.

Food availability and environmental temperature experienced by the parental generation are known to alter parental investment, with performance consequences for subsequent generations [30,31]. Poor parental condition may elicit an adaptive response in offspring via transgenerational plasticity, and offspring from parents exposed to low food may suppress their metabolic rate, or alter energy allocation towards maintenance or growth, to compensate for lower energy provisioning from the mother. Alternatively, investment in offspring can be the direct result of parental condition transfer effects, which can be adaptive, but are not contingent on environmental predictability across generations [43]. Regardless of the source of offspring trait variation, the implications of intergenerational plasticity are likely to be context dependent. For example, warmer temperatures increase the metabolic rates of ectotherms and may thereby exacerbate the fitness consequences of variation in energy acquisition and allocation in low resource environments [44]. Food availability in the parental generation is likely to alter maternal energy allocation (e.g. offspring size and composition and/or number) towards offspring as well as mediate the physiology of the offspring; the same is true for environmental temperatures in the case of ectotherms. However, it remains so far unclear as to the direction of these responses, whether they are under selection and whether they constitute an adaptive parental strategy to maximize offspring fitness.

**Figure 1.**
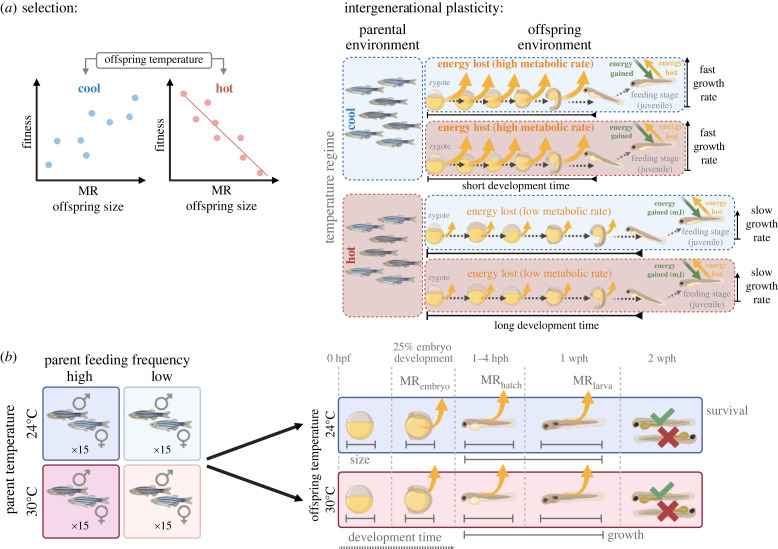
(*a*) Conceptual diagram: predicted responses of temperature-dependent selection on and intergenerational plasticity of embryo metabolic rates (*MR*) at cool and hot offspring temperatures. We hypothesise that hot offspring temperatures will select for lower metabolic rates, while selection at the cool offspring temperature will be relatively relaxed (positive but no significant correlation between fitness and metabolic rate shown). If intergenerational plasticity aligns with selection when environments across generations match, then similar trends in the direction and strength of selection should be observed. We therefore predict that parents in the warm environment (pink) will produce offspring with lower metabolic rates (smaller curved arrows) compared to parents from the cool temperature (blue), and that this will be correlated with development time and growth rates, with fitness benefits for offspring. (*b*) Experimental design: parents were held under one of four treatment combinations: 24°C or 30°C and low or high feeding frequency, then bred to produce offspring that were reared at either 24°C or 30°C. Embryo size (diameter, area, mass) and yolk area were measured at 1–4 h post fertilization (hpf), and metabolic rates (measured as rate of oxygen consumption) measured at three stages: 25% of embryonic development (*MR*_embryo_), 1–4 h post-hatching (hph; *MR*_hatch_), and 1 week post-hatching (1 wph; *MR*_larva_). Offspring were then monitored for survival up to two weeks post-hatching.

Despite evidence that metabolic rates are under selection, it is yet to be established whether parents can modify the metabolic rates of their offspring in adaptive ways. Recent work on ectotherms has shown evidence for both the presence [45,46] and absence [47] of transgenerational responses of metabolic rates to temperature. However, offspring fitness in these studies was measured indirectly as growth [46,47] or aerobic scope [45], which may trade off with actual fitness, and in some treatments [45] showed extensive mortality, hence results may be due to selective mortality. Formal tests of whether transgenerational plasticity aligns with selection on offspring metabolic rates, via measures of offspring metabolism and fitness under different environments, are currently lacking. Further, it is often unclear how selection on metabolic rates may be mediated by its correlation with traits that set the pace of life, such as developmental and growth rates. Here we manipulate parental food availability and temperature in zebrafish to determine whether context-dependent selection on offspring metabolic rates is in line with intergenerational plasticity on metabolic rates and traits that set the pace of life. We hypothesize that warm offspring temperatures will select for smaller embryo size and lower metabolic rates, while selection at the cool (benign) offspring temperature will be relaxed. Shifts in parental investment should mirror selection on offspring phenotype when their environments match—thus parents in a warm environment should produce smaller offspring with lower metabolic rates compared to parents from a cool temperature (figure 1*a*). Further, we predict that adaptive parental effects should be exaggerated when parental food availability is low, under which conditions parents should produce offspring with lower metabolic rates than parents from the high food availability environment.

## 2. Material and methods

### (a) Parent maintenance and treatments

All procedures were approved by the University of Sydney Animal Ethics Committee (protocol number: 2021/1932). Adult zebrafish were obtained from a commercial supplier (Livefish, Childers, Queensland, Australia) and housed in a controlled temperature room (22°C with 12L : 12D). The supplier maintained zebrafish at 22–24°C in large communal ponds so the parental fish were unlikely to have been closely related. The experiment was run in two replicate blocks, one month apart. Within each block, fish were first allocated randomly across four 35 l tanks (35–38 fish per tank) for two weeks to acclimate. Fish were then sexed as per [48] and 60 females and 60 males were allocated evenly across 12 experimental (11 l) tanks; each tank was filled with aged water and contained a sponge filter and a plastic plant. We conducted four parental treatments (with three replicate tanks each) in a fully factorial design (figure 1*b*). Parents were held at either 30°C or 24°C temperature, referred to hereon as ‘high’ and ‘low’ parent temperature, respectively, and either a high feeding frequency (three times per day, five days per week) or low feeding frequency (once per day, 4 days per week). Previous studies have shown that 30°C is higher than optimal, and that the low food regime was sufficient to allow growth but at a submaximal level [49,50]. The 24°C treatment represents a relatively low but benign temperature previously shown to facilitate normal growth [51].

To validate the feeding treatments used, measures of parent body mass and length taken at the end of the experiment were used to assess condition [52]. Parents were weighed (to the nearest 0.001 g) and total body length measured (to the nearest 0.1 cm), and the exponent for the slope of ln(length) and ln(mass) calculated as 2.79. Measures of body condition were then calculated as mass/length^2.79^. To maintain fish in stable temperature treatments, tanks were held within water baths, containing three submersible heaters (Aqua One 200 W; Techden, Sydney, Australia) and a powerhead water pump (Aqua One maxi). Temperature loggers recording every 15 min were placed into two tanks per temperature treatment. Tanks were maintained within ±1.5°C of their target temperature for the duration of the treatment. Fish were fed flake food (5 mg per fish; Supervit Fish Flakes, Tropical, Chorzów, Poland) [49] at each feeding event according to the regime described above and at randomized times between 8am–8pm each feeding day. A 50% water change was conducted twice per week. The adult food and temperature treatments were applied for eight weeks, after which adult fish were bred.

The evening before breeding, all fish from each replicate tank were transferred into 10 l plastic breeding tanks containing a coarse mesh base, through which fertilized eggs could pass to avoid being eaten by adults. Maintaining males and females in the same tank promotes the release of pheromones that stimulate ovulation and oviposition in females and spawning by males [53]. The next morning, breeding tanks were inspected within 1 h post-fertilization, and eggs were filtered through a sieve onto a Petri dish containing buffered E3 medium as per standard procedure for embryo rearing [54]. Unfertilized eggs or dead embryos were immediately removed.

### (b) Embryo and yolk size measurements, treatments and rearing

Within one hour of collection from parental tanks, individual fertilized embryos were sampled by sifting gently through a sieve, then photographed at the sphere stage under a dissecting microscope (×30 magnification; Leica S9D stereomicroscope with FLEXACAM C3 camera). Developmental stages of *D. rerio* are easily identifiable due to the transparency of embryos. The sphere stage shows a flat border between the blastodisc and yolk, and total embryo area and yolk area were measured to the nearest μm^2^. The ratio of yolk area to total embryo area was consistent among treatments (electronic supplementary material, figure S2). Hence, assuming that density of embryo tissue did not change with embryo size, we calculated embryo mass (μg) from embryo diameter at the sphere stage using a relationship previously determined for *D. rerio* [50]. Embryos were then placed individually into wells of 24-well culture plates containing E3 medium. For each of the four parental treatment combinations, 72 embryos were randomly allocated to each of two offspring temperature treatments (24°C and 30°C), resulting in a total of 576 embryos equally divided across 8 treatment groups: two parental temperatures (24°C versus 30°C) × two parental conditions (low versus high) × two offspring temperatures (24°C versus 30°C) (figure 1*b*). Offspring were maintained in incubators (Eurotherm Micro Digital Control Model i-80, Steridium, Australia) on a 12L : 12D light cycle for the remaining duration of the experiment. Since offspring were placed into their treatment temperatures within 3 hours of fertilization (approx. 2–4% of their total development time), we were able to separate the effect of parental from offspring environment.

### (c) Offspring metabolic rate measures

The rate of oxygen consumption ($\dot{V}$O_2_) was measured as a common proxy for metabolic rate (*MR*) of the offspring at three developmental stages: 1) 25% through embryonic development (14 and 30 h post-fertilization (hpf) for embryos incubated at 30°C and 24°C, respectively, 2) 1–4 h post-hatching (hph) and 3) one week post-hatching (wph), hereon referred to as *MR*_embryo_, *MR*_hatch_ and *MR*_larva_, respectively. Individual offspring of known identification were photographed to measure diameter (*MR*_embryo_) or length (*MR*_hatch_, and *MR*_larva_) to the nearest *μ*m, then placed into individual 80 µl (*MR*_embryo_ and *MR*_hatch_) or 500 µl (*MR*_larva_) glass vials containing Milli-Q water and a nonconsumptive O_2_ sensor spot. We used two 24-channel PreSens sensor dish readers (SDR2, PreSens, Germany), each with 24-chamber glass microplates (Loligo Systems Aps, Tjele, Denmark) to measure $\dot{V}O_{2}$ in 40 offspring and four blank vials simultaneously over a 2-h interval at their respective treatment temperature (24°C or 30°C). For a detailed description of methods, see [50]. To calculate the most linear rates of decrease in oxygen concentration within each time series dataset (adjusted for background oxygen extraction), we used the *RespR* package, designed for processing closed chamber aquatic respirometry data in R [55]. Slopes were then converted into rate of oxygen consumption, accounting for oxygen solubility of 5.91 ml O_2_ at 24°C and 5.29 ml O_2_ at 30°C (0 ppt salinity) [56].

### (d) Offspring hatching time and survival measures

Eggs were held in their individual wells of the culture plates to allow recording of embryo development time (time in hours from fertilization until hatching; hpf) and survival; their water was changed daily using a solution of Milli-Q water with 0.5 g l^−1^ of red sea salt at the treatment temperature. Based on hatching time pilot data, we monitored embryos every 2 hours from 30 hpf at 30°C and 90 hpf at 24°C until all embryos were recorded as either hatched or deceased. Within two hours of hatching, larvae were photographed for measures of larval length (0 hph) and moved into larger 6-well culture plates filled with fresh water and placed back into incubators at their respective treatment temperature. At four days post-hatching (dph), once feeding structures were fully formed, offspring were fed paramecium (4–5 dph), egg yolk (5–14 dph), flake food (5-14 dph) and *Artemia* sp. (from 15 dph) ad libitum. Larvae were measured again at one week post hatchling (1 wph) to obtain measures of growth rate (mm day^−1^ = (length at 1 wph / length at 0 hph) / 7). Larvae were monitored for survival daily until two weeks post-hatching. Sample sizes for all measures are provided in electronic supplementary material, table S1.

### (e) Analysis of parent and offspring treatment effects on parent and offspring phenotypes

All analyses were conducted in R v.4.2.3 [57]. Linear mixed effects models using the ‘lmer’ function within the *lme4* package [58] were used to analyse the effect of parental environment (feeding frequency and temperature) on parent body condition. The effect of feeding frequency (low/high), temperature (24°C/30°C) and their interaction on body condition was tested, as well as the random effect of ‘Tank ID’ within block (three per treatment). We also used linear mixed effects models to analyse the effect of parental condition (low/high feeding frequency), parental temperature (24°C/30°C), offspring temperature (24°C/30°C) and all interactions on offspring phenotypes. The significance of parent ‘Tank ID’ as a random effect was tested for all responses. We focussed on four key offspring traits: 1) embryo mass (parental investment), 2) metabolic rates (*MR*_embryo_, *MR*_hatch_ and *MR*_larva_), 3) development time (time from fertilization until hatching) and 4) growth rate (length at two weeks post-hatching divided by length at hatching). All candidate models for offspring responses are provided in electronic supplementary material, table S2. Embryo mass (μg) was included as a covariate in metabolic rate and development time models (m2 and m3; electronic supplementary material, table S2). We used embryo mass since we only had estimates of length and area for larvae at hatching and one week post-hatching, and have previously shown this to be an important indicator for hatch and larval mass [59]. We used Akaike Information Criteria (AIC) for model ranking and averaged models with *Δ* conditional AIC (AICc) < 2 using the R package *MuMin* [60–62] (electronic supplementary material, table S3) and the estimated marginal means from the best-fitting model were used for all *post-hoc* comparisons using the *emmeans* package [63]

### (f) Correlations between developmental and growth rates with metabolic rates

To explore within-individual associations among measures of developmental and growth rates with metabolic rates, we ran repeated measures correlations using the package *rmcorr* [64]. Using a repeated measures framework accounts for the non-independence of observations measured on the same individuals.

### (g) Selection analysis

We used a classic multiple regression approach derived from evolutionary theory to characterize temperature-dependent selection acting on embryo metabolic rates, within each parental environment [42]. This framework allows for standardized and comparable estimates of both linear (*β*) and nonlinear (*γ*) selection coefficients. For each form, we estimated the direction (sign of coefficients) and strength (magnitude of coefficients) of selection acting on offspring mass and mass-independent metabolic rate (*MIMR*), across incubation temperatures, as per [42]. These measures have been used previously to provide a more complete picture of the fitness landscape for offspring metabolic rates [9].

Fitness was measured as survival from fertilization to two weeks post-hatching. This period of life typically shows greatest mortality rates in egg-laying fish and is considered a bottleneck to reproduction, and therefore fitness [65]. Survival was treated as binary data—offspring that survived to two weeks post-hatching were assigned ‘1’, whereas offspring that died before two weeks were assigned ‘0’. First, autocorrelation between traits was checked to determine which traits should be included in the analysis. Metabolic rates at each ontogenetic stage were significantly correlated (when embryo mass was included as a covariate; *F*_3,1724_ = 3434, *p* < 0.0001), particularly between *MR*_embryo_, and *MR*_hatch_ (*r*^2^ = 0.71). We decided to use mass-independent metabolic rate (*MIMR*) since recent work has shown that including both mass and metabolic rate in selection analyses can overestimate the strength of selection on metabolic rates [66]. Correlations between embryo mass, *MIMR*_embryo_, and *MIMR*_larva_ were relatively weak and variance inflation factors were less than 5, hence both *MIMR*_embryo_, and *MIMR*_larva_ were included, but *MIMR*_hatch_ was excluded from the analysis. To prepare data for selection analysis, we followed the method of [42]: first, within each combination of parent and offspring treatment, we converted predictor variables of embryo mass, *MIMR*_embryo_ and *MIMR*_larva_ into units of standard deviation (mean of 0, standard deviation of 1) and divided each measure of absolute fitness by mean absolute fitness to mean-centre survival.

Survival data were fitted using logistic regression in a generalized linear model using the ‘glm’ function. We ran a series of nested models to test for differences in linear and nonlinear forms of selection. We first tested whether there were significant differences in selection among parental and offspring environments, via a sequential model-fitting method [67,68]. We then tested for significant interactions between selection (linear and nonlinear) and environment (parental condition, parental temperature and offspring temperature). Since we only found significant interactions between selection and offspring temperature, fitness data were mean-centred (see details above) within offspring temperature and selection coefficients were estimated for offspring incubated at 24°C and 30°C separately. Selection coefficients from the logistic regression were transformed into linear estimates as per [69]. Following [70], we doubled quadratic regression coefficients and their standard errors before reporting selection gradients.

## 3. Results

### (a) Effects of parental environment on parent body condition and offspring size

**Figure 2.**
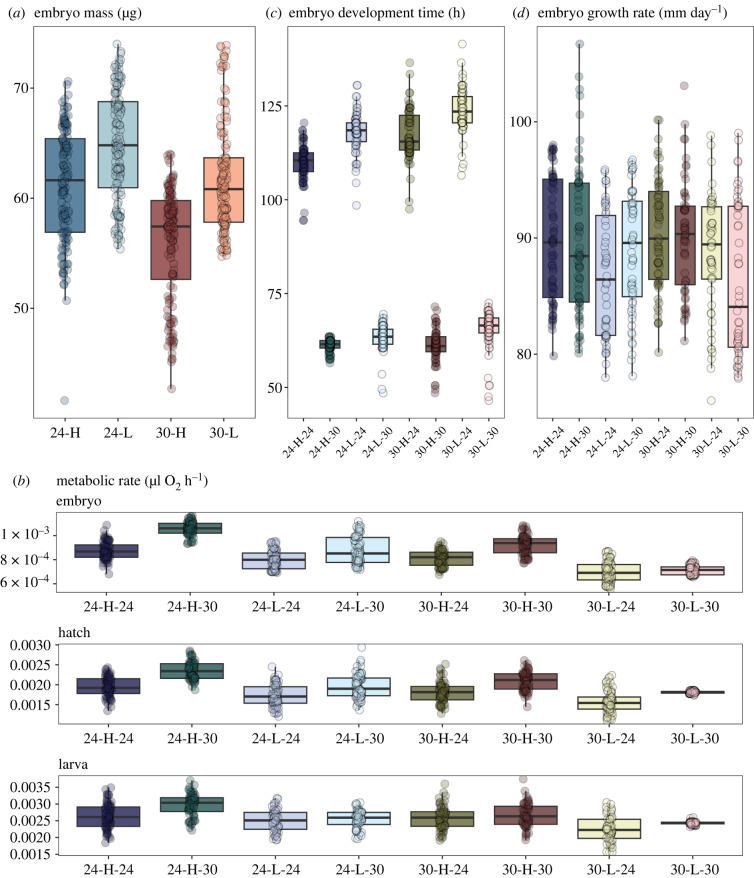
Offspring phenotypes in response to parent and offspring treatments. Responses of (*a*) embryo mass, (*b*) metabolic rate (*MR*_embryo_, *MR*_hatch_, *MR*_larva_), (*c*) development time and (*d*) growth rate, measured across combinations of parent temperature (24°C or 30°C), feeding frequency (low; ‘L’ or high; ‘H’) and offspring temperature (24°C or 30°C). The first number in a treatment description refers to parent temperature and the second refers to offspring temperature. Note that metabolic rates shown in panel (*b*) do not account for the significant effect of embryo mass.

Parents in the low feed treatment showed significantly lower body condition than individuals within the high feed treatment (*t* = −6.44, d.f. = 7.34, *p* < 0.001), however, there were no differences in condition between high- and low-temperature treatments (electronic supplementary material, figure S1). Despite low body condition, parents held under the low-feed frequency regime produced embryos that were heavier than those from high-condition parents (table 1). Although there appeared to be a trend for heavier offspring from cool-reared parents (figure 2*a*), there was no significant effect of parent temperature on embryo mass (table 1).

**Table 1.** Output from best-fitting linear mixed effects models. Estimates provided for fixed effects of parent (P) temperature (24°C or 30°C), parent feeding frequency (high; H or low; L) and offspring (O) temperature (24°C or 30°C) on offspring phenotypes: 1) embryo mass, 2) metabolic rates (2a. *MR*_embryo_, 2b. *MR*_hatch_, 2c. *MR*_larva_), 3) development time, 4) growth rate and 5) survival to two weeks post hatching. For survival, logistic generalized linear mixed effect regression was used and individuals were assigned either ‘1’ for alive at two weeks post-hatching or ‘0’ for dead. Parental tank ID was included as a random effect in all models. All candidate models are provided in electronic supplementary material, table S2 and ranked in electronic supplementary material, table S3. All comparisons are made in relation to ‘L’ parent feed frequency and 30°C parent and offspring temperature. Significance level set at *p* < 0.05.

predictors	estimate	s.e.	d.f.	*t*-value	*p*-value
1. Embryo mass					
intercept	56.70	1.49	15.03	38.12	<0.0001***
P feed (L)	8.45	1.06	298.95	7.98	<0.0001***
P temperature (30)	0.18	1.26	233.14	0.14	0.89
P feed (L) × P temperature (30)	0.37	1.16	367.29	0.25	0.81
2a. Log_10_ *MR*_embryo_					
intercept	−4.27	0.08	184.05	−56.04	<0.0001***
log_10_ Embryo mass	0.69	0.04	174.21	16.02	<0.0001***
P feed (L)	−1.01	0.11	340.08	−9.30	<0.0001***
O temperature (30)	0.06	0.00	505.68	25.81	<0.0001***
P temperature (30)	−0.03	0.00	13.88	−6.02	<0.0001***
log_10_ embryo mass × P feed (L)	0.51	0.06	333.03	8.27	<0.0001***
2b. Log_10_ *MR*_hatch_					
intercept	−4.00	0.13	105.98	−30.44	<0.0001***
log_10_ embryo mass	0.72	0.07	103.55	9.79	<0.0001***
O temperature (30)	0.07	0.00	442.76	17.79	<0.0001***
P feed (L)	−0.78	0.20	181.16	−3.93	<0.001**
P temperature (30)	−0.01	0.01	12.80	−2.48	0.03
log_10_ embryo mass × P feed (L)	0.38	0.11	179.19	3.43	<0.001***
2c. Log_10_ *MR*_larva_					
intercept	−3.19	0.14	198.41	−22.60	<0.0001***
log_10_ embryo mass	0.35	0.08	187.11	4.38	<0.0001***
O temperature (30)	0.03	0.01	370.10	6.09	<0.0001***
P feed (L)	−0.06	0.01	14.94	−8.31	<0.0001***
P temperature (30)	−0.02	0.01	9.29	−2.75	0.02*
3. Development time					
intercept	110.66	0.96	24.89	115.63	<0.0001***
O temperature (30)	−48.77	0.82	564.05	−59.61	<0.0001***
P feed (L)	6.79	1.17	76.00	5.79	<0.0001***
P temperature (30)	6.24	1.24	55.30	5.03	<0.0001***
O temperature (30) × P feed (L)	−5.72	1.19	548.31	−4.80	<0.0001***
O temperature (30) × P temperature (30)	−6.49	1.16	565.52	−5.59	<0.0001***
P feed (L) × P temperature (30)	0.65	1.54	124.97	0.42	0.68
O temperature (30) × P feed (L) × P temperature (30)	2.40	1.81	486.64	1.32	0.19
4. Growth rate					
intercept	89.78	0.65	460	138.73	<0.0001***
O temperature (30)	−0.10	0.93	460	−0.10	0.92
P feed (L)	−3.04	1.01	460	−3.02	0.003**
P temperature (30)	0.57	0.95	460	0.60	0.55
O temperature (30) × P feed (L)	2.32	1.41	460	1.64	0.10
O temperature (30) × P temperature (30)	−0.12	1.37	460	−0.09	0.93
P feed (L) × P temperature (30)	1.37	1.43	460	0.96	0.34
O temperature (30) × P feed (L) × P temperature (30)	−4.50	2.03	460	−2.22	0.02*
5. Survival					
intercept	1.08	0.20		5.39	<0.0001***
O temperature (30)	−0.104	0.21		−0.49	0.63
P feed (L)	−0.72	0.21		−3.42	<0.001***
P temperature (30)	−0.08	0.22		−0.37	0.71
O temperature (30) × P temperature (30)	0.16	0.35		0.48	0.66
P feed (L) × P temperature (30)	−0.14	0.36		−0.40	0.69
O temperature (30) × P feed (L	0.11	0.36		0.32	0.75

### (b) Effects of parental and offspring environments on offspring metabolic rates

**Figure 3.**
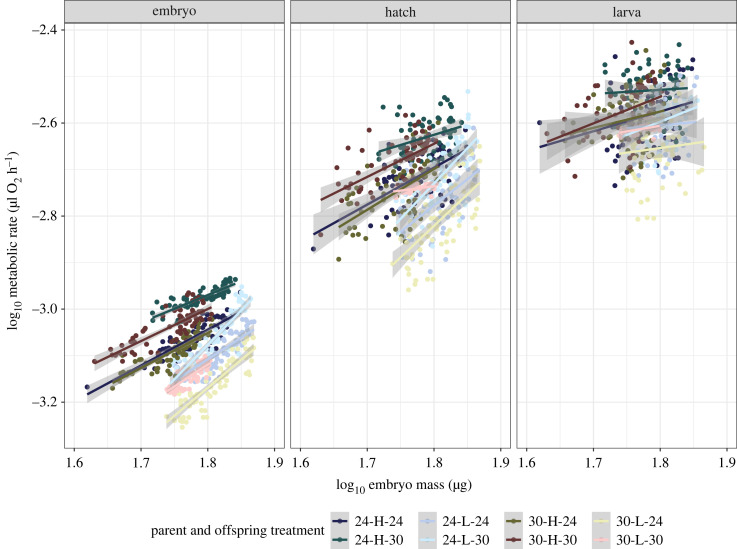
The relationship between offspring metabolic rates (log_10_ *MR*_embryo_, log_10_ *MR*_hatch_, log_10_ *MR*_larva_) and log_10_ embryo mass in response to parent and offspring treatments. Coloured data points and lines reflect parent temperature (24°C or 30°C), feeding frequency (low; ‘L’ or high; ‘H’), and offspring temperature (24°C or 30°C). Grey bars are standard error. The first number in a treatment description refers to parent temperature and the second refers to offspring temperature.

Offspring reared at the high-temperature treatment showed significantly higher metabolic rates than those reared at the low-temperature treatment (table 1, figure 2*b*). We also found significant parental environment effects on offspring metabolic rates (table 1, figure 2*b*). Parents exposed to the high-temperature or low-food treatments produced offspring with lower metabolic rates at embryo, hatch and larval stages. We also found a significant interaction between embryo mass and parent feed frequency, where the slope between embryo metabolic rate and mass was steeper in offspring from low-feed frequency parents (table 1, figure 3).

### (c) Effects of parental and offspring environments on offspring developmental and growth rates

Offspring incubated at the low temperature took almost twice as long to develop than those incubated at the warm temperature (table 1, figure 2*c*). More interestingly, development time at a given offspring temperature was affected by the parental temperature as well as the parental feed frequency, being extended in offspring from low-feed frequency or high-temperature parents; thus hatching was delayed by 9 h on average when offspring reared at the cool temperature came from low-feed compared with high-feed parents (electronic supplementary material, table S1, figure 2*c*). We also found significant interactive effects between offspring temperature and parent feed treatment, and between offspring temperature and parent temperature on development time. High food treatment parents produced offspring that developed faster when reared at the low offspring temperature, but not high offspring temperature treatment (table 1, figure 2*c*, *t* = −2.34, *p* = 0.088). Embryos developing in the cool treatment developed faster when their parents were also from the cool temperature, relative to parents from the warm temperature (table 1, figure 2*c*, *t* = −0.90, *p* = 0.807).

Larval growth rates during the second week post-hatching were faster in offspring from high-feed parents (table 1, figure 2*d*). We also found a significant three-way interaction between parental temperature, parent condition and offspring temperature for larval growth rate: growth was slowest in offspring from the low offspring temperature treatment and when parents were from both the low feeding frequency and the low temperature (table 1).

### (d) Correlations between offspring traits

**Figure 4.**
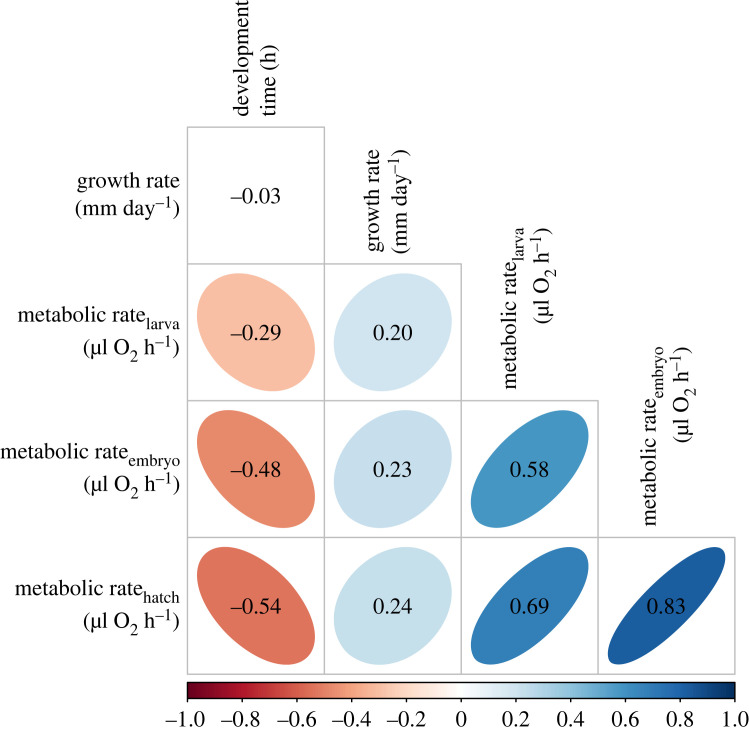
Correlation plots for offspring phenotypes. Pairwise correlations between offspring traits: metabolic rates, embryo development time and larval growth rate, across combinations of parent and offspring treatments. Coloured plots represent significant correlations between traits.

We found significant positive correlations between all metabolic rates (embryo, hatch, larval) and between larval growth rates and these three metabolic rates (figure 4). In contrast, embryo development time was significantly negatively correlated with metabolic rates. We found no significant correlation between embryo development time and larval growth rate overall, however they were significantly negatively correlated within offspring temperature treatments (electronic supplementary material, figure S3).

### (e) Effects of parental environment on offspring survival

Overall, we found that survival was lowest in offspring from parents in the low-feed frequency regime, but offspring and parent temperatures showed no effect on offspring survival to two weeks post hatching (table 1). Although parents in the low-feed treatment produced larger offspring, embryo mass did not itself predict survival.

### (f) Selection on offspring metabolic rates

**Figure 5.**
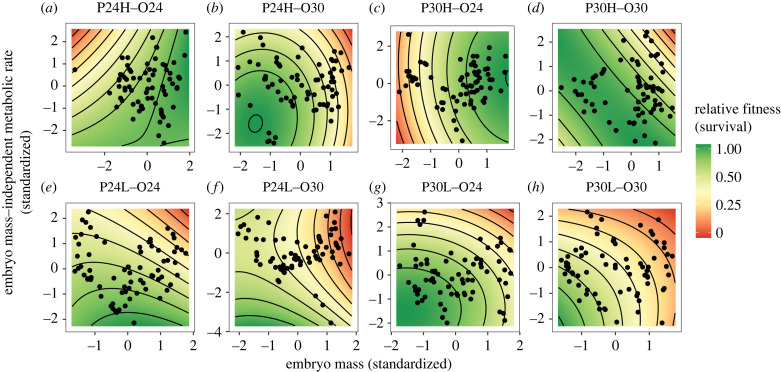
Selection surface plots. Selection on embryo mass (μg) and metabolic rate (*MR*_embryo_; μlO_2_h^−1^) across combinations of parent (P) temperature (24°C or 30°C) and feeding frequency (high; H or low; L) and offspring (O) temperature (24°C or 30°C) environments.

Offspring from low-food parents showed greater survival when they had relatively low embryo metabolic rates, as shown by significant negative directional selection (table 2, figure 5*e–h*). Across all offspring high-temperature treatments, we found evidence for negative directional selection on embryo metabolic rates (table 2, figure 5*b,d,f,h*). We also found positive directional selection on offspring embryo mass when they were reared at the low temperature from high-feed parents (P24HO24 and P30HO24; figure 5*a*,*c*) or they were reared at the high-temperature but from high-feed and low-temperature parents (P24HO30; figure 5*b*). Conversely, we found negative directional selection on embryo mass when offspring originated from parents reared at the high-temperature and low-food treatments (P30LO24 and P30LO30; figure 5*g,h*). There was also evidence for stabilizing selection on embryo metabolic rate in P30LO24 (figure 5*g*), as shown by a significant negative quadratic coefficient (table 2). We found no significant directional selection on larval metabolic rates, however there was significant positive correlational selection for embryo and larva metabolic rates in P24LO30 (table 2, figure 5*f*), suggesting that consistently lower metabolic rates were favoured in this environment.

**Table 2.** Selection coefficients (mean and standard error). Direction and strength of linear (*β*) and nonlinear (*γ*) selection on embryo mass and mass-independent metabolic rates across two life stages (*MIMR*_embryo_ and *MIMR*_larva_; μlO_2_h^−1^) in *Danio rerio*. Fitness was measured as survival to two weeks post hatching. Results shown for each combination of parent (P) temperature (24°C or 30°C), parent feeding frequency (high, H or low, L) and offspring (O) temperature (24°C or 30°C). Significant selection gradients (*p* < 0.05) shown in bold.

parent environment	offspring environment		*β*	*γ*		
				embryo mass	*MIMR*_embryo_	*MIMR*_larva_
P24H	O24	embryo mass	**0.134** (**0.041)**	−0.053 (0.114)	0.010 (0.055)	−0.020 (0.043)
		*MIMR*_embryo_	−0.061 (0.041)		−0.054 (0.092)	0.082 (0.048)
		*MIMR*_larva_	−0.013 (0.033)			0.011 (0.075)
				embryo mass	*MR*_embryo_	*MR*_larva_
	O30	embryo mass	**0.039** (**0.036)**	−0.117 (0.105)	0.052 (0.057)	−0.030 (0.060)
		*MIMR*_embryo_	**−0.157** (**0.040)**		0.051 (0.113)	−0.084 (0.065)
		*MIMR*_larva_	−0.055 (0.035)			0.181 (0.095)
				embryo mass	*MR*_embryo_	*MR*_larva_
P30H	O24	embryo mass	**0.180** (**0.056)**	0.371 (0.224)	0.040 (0.125)	0.004 (0.129)
		*MIMR*_embryo_	0.052 (0.033)		0.002 (0.068)	0.056 (0.079)
		*MIMR*_larva_	−0.002 (0.041)			−0.179 (0.119)
				embryo mass	*MR*_embryo_	*MR*_larva_
	O30	embryo mass	−0.109 (0.089)	0.210 (0.215)	−0.165 (0.181)	0.181 (0.258)
		*MIMR*_embryo_	**−0.167** (**0.074)**		−0.091 (0.074)	0.320 (0.218)
		*MIMR*_larva_	−0.021 (0.045)			0.100 (0.073)
				embryo mass	*MR*_embryo_	*MR*_larva_
P24L	O24	embryo mass	−0.092 (0.070)	−0.106 (0.166)	−0.083 (0.124)	−0.160 (0.095)
		*MR*_embryo_	**−0.122** (**0.047)**		0.346 (0.281)	0.157 (0.138)
		*MR*_larva_	0.046 (0.059)			0.090 (0.159)
				embryo mass	*MR*_embryo_	*MR*_larva_
	O30	embryo mass	−0.200 (0.057)	−0.343 (0.270)	−0.329 (0.222)	0.043 (0.099)
		*MR*_embryo_	**−0.070** (**0.035)**		0.329 (0.320)	**0.297** (**0.149)**
		*MR*_larva_	−0.050 (0.037)			−0.060 (0.093)
				embryo mass	*MR*_embryo_	*MR*_larva_
P30L	O24	embryo mass	**−0.122** (**0.056)**	−0.039 (0.113)	0.038 (0.095)	0.077 (0.086)
		*MR*_embryo_	**−0.161** (**0.054)**		**−0.353** (**0.138)**	−0.038 (0.093)
		*MR*_larva_	−0.017 (0.038)			−0.051 (0.081)
				embryo mass	*MR*_embryo_	*MR*_larva_
	O30	embryo mass	**−0.166** (**0.056)**	0.218 (0.233)	0.171 (0.111)	−0.173 (0.125)
		*MR*_embryo_	**−0.120** (**0.042)**		−0.168 (0.104)	−0.008 (0.065)
		*MR*_larva_	0.022 (0.030)			0.144 (0.120)

## 4. Discussion

Intergenerational effects can be an important source of offspring phenotypic variation—here we provide evidence of adaptive intergenerational plasticity for offspring metabolic rates. We found that low parental food availability negatively impacted offspring survival, but also altered offspring metabolic phenotypes in a direction that aligned with selection on offspring traits. The low feeding frequency treatment in our study produced low-condition parents that invested in larger offspring, compared with parents from the high feeding frequency treatment. We also found that when parents were reared under either the warm (30°C) temperature, low feeding frequency treatment or both, they produced offspring with lower metabolic rates. Warm developmental temperatures generally increase the metabolic rates of offspring; however, we show that at these temperatures selection acts to decrease offspring metabolism, and that parents modify their offspring accordingly.

### (a) Parental condition and offspring temperature increased selection on offspring metabolism

Overall, we found that low parental food levels increased the presence and strength of selection acting on embryo metabolic rate (*MR*_embryo_), such that offspring with lower MR_embryo_ were more likely to survive a critical period of early development (compare figure 4*e–h* with *a–d*). Previous work has clearly demonstrated the direct effects that environmental temperature and food availability produce on metabolic rates [21,24,71,72]. Acute effects of warming generally increase metabolic rates in ectotherms, yet acclimatization or adaptation can act to suppress energy expenditure [73,74]. Similarly, low food availability often selects for reduced metabolic rates [75], presumably to conserve energy reserves. Further, temperature and food availability can interact to affect metabolism in complex ways, with evidence for temperature mediating both an increase and decrease in metabolism with increases in food availability [76–78].

### (b) Intergenerational plasticity is adaptive when environments are consistent across generations

We found similar patterns between intergenerational plasticity and selection on offspring metabolic rates when parent and offspring temperatures matched. Parents reared under the warm temperature treatment produced offspring with lower metabolic rates, which were more likely to survive than warm-reared offspring from cool-reared parents. Consequently, offspring with slower metabolic rates showed greater survival in warm developmental temperatures, particularly when they originated from parents from the low food treatment. The downregulation of offspring metabolism is likely to be particularly crucial when food availability is low, where offspring are more likely to be reliant on internal energy reserves to fuel early life growth, maintenance and development. The alignment of intergenerational plasticity and selection provides evidence that shifts in offspring metabolic phenotypes can be adaptive when the environment in the parent generation matches that of the offspring generation. This has often been assumed in studies measuring performance metrics, such as growth or aerobic capacity, which may trade off with actual fitness [31,39,79]. Through use of a selection analysis, our study provides standardized, comparable estimates of selection, showing that parents can programme their offspring with metabolic phenotypes that enhance early life survival. Our findings, however, have worrying implications for environmental mismatches between generations. We acknowledge that our study was conducted on zebrafish reared under stable laboratory conditions, and that wild-caught fish or other taxa may respond differently [80]. However, under increasingly warmer and more variable climates, parents may not be able to keep pace with provisioning their offspring to enhance survival during a vulnerable life stage, and there may be increasing reliance on thermal acclimation to buffer populations to environmental change.

### (c) Potential proximal mechanisms underlying intergenerational effects on offspring metabolism

Metabolic suppression as a means to conserve energy has been well documented, yet intergenerational mechanisms are less well explored [24,73,74]. Across generations, epigenetic mechanisms such as changes in DNA methylation can facilitate developmental thermal plasticity to buffer offspring from stressful temperatures [81–83]. One clear mechanism by which parents may alter the transgenerational thermal sensitivity of offspring metabolic and life-history traits is through changes in the density and efficiency of mitochondria [46]. Fasting and warm temperature regimes can enhance mitochondrial efficiency, such that a greater amount of ATP is produced per amount of oxygen consumed [49]. For species that provision their offspring with finite energy reserves in eggs, energy-demanding warm temperatures may elicit an adaptive response in parents to produce energy-efficient offspring. It may be that parents can programme their offspring with more efficient mitochondria to compensate for a predicted energetically costly environment, as reflected by lower metabolic rates [46]. We found that metabolism until two weeks post-hatching was unrelated to growth rates, supporting previous work finding that these two rates can be decoupled and that low metabolic rates do not necessitate slow growth rates because it is mitochondrial efficiency rather than metabolic rate *per se* that determines availability of ATP for growth [84]. While fitness benefits of reduced metabolism were observed within this study, trade-offs with oxidative stress may manifest later in life, affecting fitness-enhancing processes [85]. While our study did not detect any negative consequences of metabolic suppression for early life survival in zebrafish, previous work has shown that slow metabolic phenotypes possess lower competitive ability, compared with fast metabolic phenotypes [9]. What is needed now is to go beyond measures of oxygen consumption to investigate the capacity for parents to alter the efficiency of ATP production in their offspring and mediate fitness under warmer and more nutrient-poor environments.

### (d) The presence and form of selection on metabolism varied across ontogeny

Despite clear evidence for selection on metabolic rate during embryonic development, we found that, across all environments, directional selection on larval metabolic rate (*MR*_larva_) was absent. A recent meta-analysis showed limited evidence for selection on metabolic rate, where the majority of selection coefficients were measured during the adult life stage [86]. Variation or flexibility in metabolic rate may confer a fitness advantage, particularly under selection regimes that change across time and space [24]. Metabolic rate is not a single trait, hence metabolic rates expressed at different life stages are likely to affect fitness to varying extents [12,22]. Metabolic rates may be repeatable, such that they are correlated across the life history, yet we found differences in selection on metabolic rates measured one week apart. In our study, we fed hatched larvae ad libitum, which may have relaxed selection on larval traits. Alternatively, it may be that there are fitness consequences for a low or high larval metabolic rate that were not measured in this study. Survival is a key component, but not an absolute measure of fitness, and further measures are needed of both metabolism across ontogeny and lifetime reproductive output. We did, however, observe negative correlational selection on embryo mass and *MR*_larva_ in offspring reared at the cool (24°C) temperature, from high condition parents also reared at 24°C. Offspring mortality was greatest in smaller embryos with relatively high *MR*_larva_, possibly because the reduced endogenous energy reserves often attributed to smaller offspring were insufficient to sustain higher metabolism in the larval stage. Variation in the strength, form, and direction of selection on combinations of early life traits across environments reveals the diversity of fitness landscapes that organisms may enter, and the challenges that parents face when matching offspring phenotype to enhance performance within a given environment.

### (e) Potential indirect selection on developmental and growth rates

We found that metabolic rates measured from the embryo stage through to one week post hatching were consistently negatively correlated with development time and positively correlated with growth rate, but that developmental and growth rates were only correlated within offspring temperature treatments. Pace-of-life theory proposes that natural selection should favour the integration of a suite of life-history and metabolic traits that together enhance fitness [87]. In our study, warm and low-condition parents produced offspring with lower metabolic rates, with evidence for a slower pace-of-life, including extended development time and reduced growth rates. Potential mechanisms underlying this response from parents include epigenetic modification such as DNA methylation in gametes or early developmental stages, or genetic constraints. Our finding that selection acts to reduce embryo metabolic rate in the warm offspring treatment may inadvertently also act to reduce the pace of life if these traits are both phenotypically and genetically, correlated. There is evidence however, that pace-of-life traits can be decoupled, whereby growth and developmental rates, for example, can evolve independently [88]. Further measures of multivariate selection will help to disentangle the underlying drivers of correlated traits related to the pace of life [89]

## 5. Conclusion

Our study shows the importance of intergenerational plasticity as a source of variation in metabolic rates during early life stages. When parent and offspring environments match, parents can programme offspring to express metabolic phenotypes that align with selection on embryonic metabolic rate. Offspring with lower metabolic rates showed greater survival when reared under warm temperatures, and this response was particularly evident when offspring originated from low-condition parents. Our findings support previous evidence that the unpredictability of offspring environment may in part explain why adaptive parental effects are not always, or only weakly, observed. However, identifying the mechanistic basis of parental effects on variation in metabolic rate is an important next step.

## Data Availability

All data and code have been made publicly available for peer review from the Open Science Framework: https://osf.io/6357s/?view_only=9c6e1ac841fb4e6188fd297aeeaa2733 [90]. Supplementary material is available online [91].
